# Properties of Wedge Wire Bonded Connection Between a Composite Copper Core Aluminum Shell Wire and an 18650 Cylindrical Lithium-Ion Battery Cell

**DOI:** 10.3390/ma17215237

**Published:** 2024-10-28

**Authors:** Krzysztof Bieliszczuk, Tomasz M. Chmielewski

**Affiliations:** Department of Joining Engineering, Faculty of Mechanical and Industrial Engineering, Warsaw University of Technology, Narbutta 85, 02-524 Warsaw, Poland

**Keywords:** wire bonding, Cucore, battery joining, solid-state welding

## Abstract

Wedge wire bonding is a solid-state joining process that uses ultrasonic vibrations in combination with compression of the materials to establish an electrical connection. In the battery industry, this process is used to interconnect cylindrical battery cells due to its ease of implementation, high flexibility and ease of automation. Wire materials typically used in battery pack manufacturing are pure or alloyed aluminum and copper. While copper wires possess better electrical properties, the force used in the bonding process can lead to cell isolator damage and cell thermal runaway. This is an unacceptable result of the bonding process and has led to the development of new types of composite wires containing a copper core embedded in an aluminum shell. This material has the advantage of high copper electrical and thermal conductivity combined with less aggressive bonding parameters of the aluminum wire. The aim of this study was to establish a process window for the wedge wire bonding of 400 µm composite copper–aluminum Heraeus CucorAl Plus wire on the surface of a BAK 18650 battery cell. This study was conducted using a Hesse Bondjet BJ985 CNC wire bonder fitted with an RBK03 bond head designed for the bonding of copper wires. The methods used in this study included light and scanning electron microscopy of bond and battery cell cross-sections, shear testing on the XYZtec Sigma bond tester system, and energy dispersive spectroscopy. The results were compared with a previous study conducted using a wire of the same diameter and made out of high-purity aluminum.

## 1. Introduction

Wire bonding is a solid-state joining process [1] based on the same principles as ultrasonic welding [2] that uses a combination of ultrasonic vibrations and pressure to form a connection between the substrate and the bonding wire. The main goal of the bonding wire is to provide an electrical connection between two or more bonds. In battery assembly, the popularity of the wire bonding process is rising due to the ease of implementation, low cost and flexibility, eliminating the need for strict tolerances of the battery pack to be maintained [3].

CucorAl PLUS bonding wires consisting of a copper core clad in an aluminum shell have been developed for the wire bonding process on the surface of Al-Si bond pads of power electronics components. In this process, bonding with copper wire is not feasible because Cu wire bonding requires much more force and ultrasonic power in comparison to Al wire bonding [4,5,6,7,8]. As a result, the copper wire sinks into softer Al metallization, leading to chip damage and poor mechanical properties of the joint [9,10]. According to Heresus, the CucoreAl PLUS wire manufacturer, their product provides improved electrical and mechanical properties in comparison with pure Al wire [11]. This has been confirmed by experimental studies, which have shown that the implementation of CucorAl wire significantly improves the lifetime of the bond in comparison with standard aluminum wire [12,13,14]. Due to the aluminum shell of the wire, the bonding does not require significantly more force and ultrasonic power and can be implemented in machines not designed to bond copper wires. In battery pack manufacturing, this contributes to a safer process that has less chance of damaging the components of the battery cell such as the internal insulator located under the battery crimp.

This study was focused on determining the feasibility of the application of the CucorAl PLUS wire in the battery pack assembly process. It is a continuation of our previous studies in the area of battery wire bonding processes [15,16] and its goal is to provide a reproducible experiment including full information on the process parameters and allow for direct comparisons of wire bonding with different wire materials.

## 2. Materials and Methods

For a more detailed description of the wire bonding process and its phases, please refer to our previous research [15]. To enhance the process repeatability, laser-cleaning with 40% of the laser power was conducted based on the results of our laser ablation cleaning study [16]. This allowed for a direct comparison of the experimental results between the Heraeus GmbH (Hanau, Germany) 400 µm AluBond Pure H11 aluminum wire and the currently used Heraeus 400 µm CucorAl PLUS aluminum-clad copper-core wire.

The bonding wire was Heraeus CucorAl PLUS, consisting of a copper core made out of high-purity copper (min. 99.99%) and high-purity aluminum cladding (min. 99.99%) [17]. The ratio of the cross-section area of the copper core to the cross-section area of the aluminum cladding was 45/55%. 

**Figure 1:**
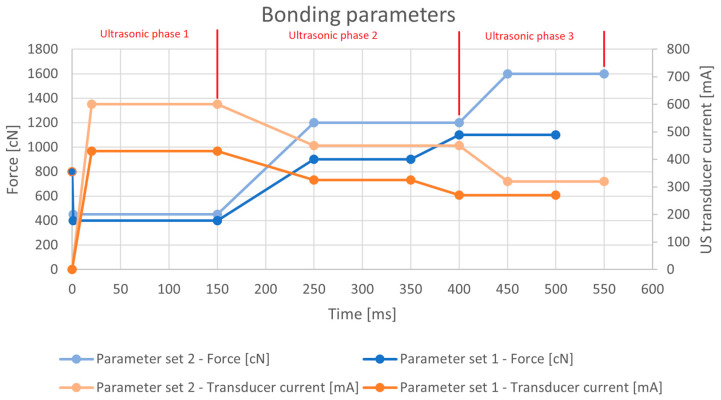
Wire bonding process parameters.

The wire bonding process in this study was composed of several phases:

- touchdown, where the sonotrode detected the height of the substrate material and applied an 800 cN pre-bonding force;
- three ultrasonic bonding phases (as seen in Figure 1), where
  ○ the first ultrasonic phase used high ultrasonic power and low force to push away any impurities from the interface area,
  ○ the second ultrasonic phase used medium ultrasonic power and medium force to introduce shearing on the material interface,
  ○ the third ultrasonic phase used low ultrasonic power and high force to stabilize the connection;
- movement of the bond head to the next bonding area.

The process was conducted for two sets of bonding parameters, each consisting of bond force and ultrasonic transducer current, as can be seen in Figure 1. The first parameters were identical to those in previous studies using high-purity aluminum wire [15,16]. The second set of parameters was chosen to achieve higher deformation of the bonding wire at around 1/3 of the wire outside diameter, as this is a level of deformation that was achieved in previous studies of the AluBond Pure H11 aluminum wire. The changes in the second set of wire bonding parameters consisted of force and ultrasonic current increases as well as an extension of the second phase time by 50 ms.

**Figure 2:**
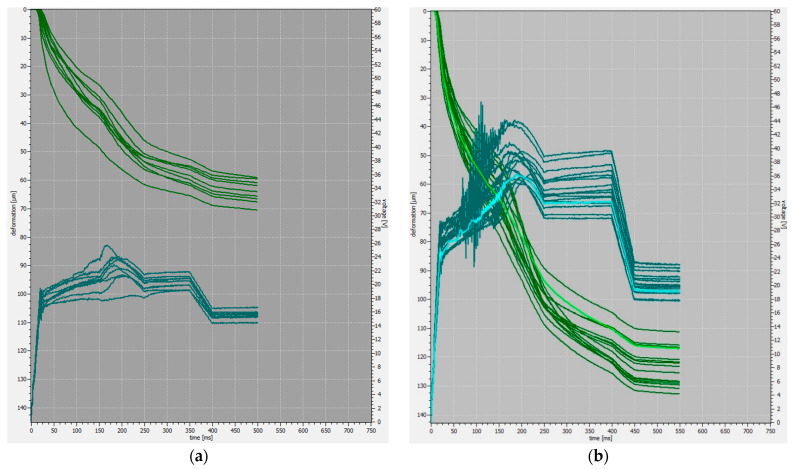
Process curves—deformation (green) and transducer voltage (blue)—for parameter sets 1 (**a**) and 2 (**b**).

The process curves associated with the first set of parameters can be seen in Figure 2a. The deformation of the wire (green) was in the range of 58–71 µm. For the second set of parameters, deformation was in the range of 110–134 µm, as can be seen in Figure 2b.

The wire bonding process was conducted on the positive terminal of BAK Battery Co., Ltd. (Shenzhen, China) N18650CP BAK A02 lithium-ion cylindrical battery cells. The material of the cell case was nickel-plated DC04 steel.

The wire bonding machine used in this study was Hesse Mechatronics GmbH (Paderborn, Germany) BJ985 with software version 5.4.15.1. The machine was equipped with an RBK03 bond head and a Hesse Mechatronics 65408-400 tungsten carbide sonotrode. The resonance frequency of the sonotrode was 58 kHz.

The cells were laser-cleaned with the ATMSolutions Sp. z o.o. Sp.k. (Łomianki, Poland) ATMS 4060 laser marking system. The cleaning parameters are presented in Table 1. Those parameters were established in our previous study, which proved that the laser cleaning process has a positive influence on wire-bonding-process stability [16]. The samples were bonded within 2 min of the laser cleaning process.

The samples for the cross-section analysis were mounted in ATM Qness GmbH (Mammelzen, Germany) DUROPLAST electroconductive hot mounting powder and ground using #80, 200, 400, 800, 1200 and 2000 silicon carbide papers. Polishing was conducted using the Struers ApS (Ballerup, Denmark) DiaPro Nap 5 µm diamond solution followed by the Struers OP-S 0.25 µm colloidal silica solution. Macroscopic images of the samples and microscopic images of the cross-sections were acquired using the Olympus (Tokyo, Japan) BX51M light microscope equipped with an Olympus DP23 camera. Scanning electron microscopy (SEM) and energy dispersive spectroscopy (EDS) were conducted using the Thermo Fisher Scientific Inc. (Waltham, MA, USA) Axia ChemiSEM microscope.

The shear testing was conducted in accordance with the DV-2811 [18] on the XYZtec bv (Panningen, The Netherlands) Sigma tester using a 1.2 mm shear tool with the test parameters in Table 2.

## 3. Results

**Figure 3:**
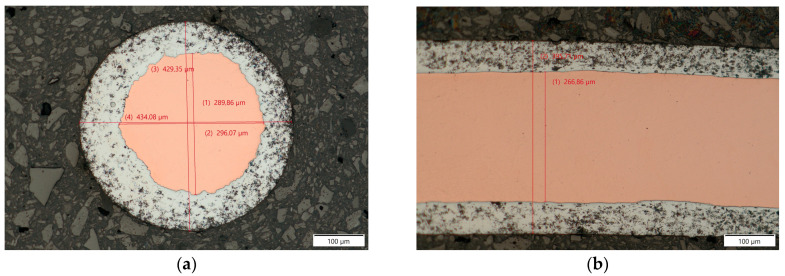
CucorAl Plus wire transversal (**a**) and longitudinal (**b**) cross-section in 200× light microscopy.

To properly interpret the changes in the wire during the wire bonding process, cross-sections of the non-bonded wire were analyzed using optical microscopy. As seen in Figure 3a, the copper core was not always centered in the aluminum cladding. The copper core cross-section did not form a uniform circle. Figure 3b shows that both the copper core diameter and the wire outside diameter were consistent throughout the longitudinal cross-section. 

**Figure 4:**
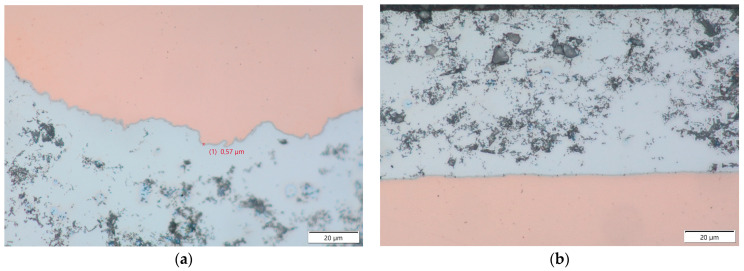
CucorAl Plus wire transversal (**a**) and longitudinal (**b**) cross-sections in 1000× light microscopy.

Images with higher magnification showed the formation of intermetallic compounds on the boundary between the copper core and the aluminum cladding, but their thickness was limited to about 0.6 µm. This was visible on both the transversal cross-section in Figure 4a as well as in the longitudinal cross-section seen in Figure 4b.

The ratio of the cross-sectional area of the copper wire to the cross-sectional area of the aluminum cladding was about 45/55%. 

The wire bond cross-sections were analyzed using light microscopy to detect defects in the bonds and compare the bonds made with two sets of different parameters.

**Figure 5:**
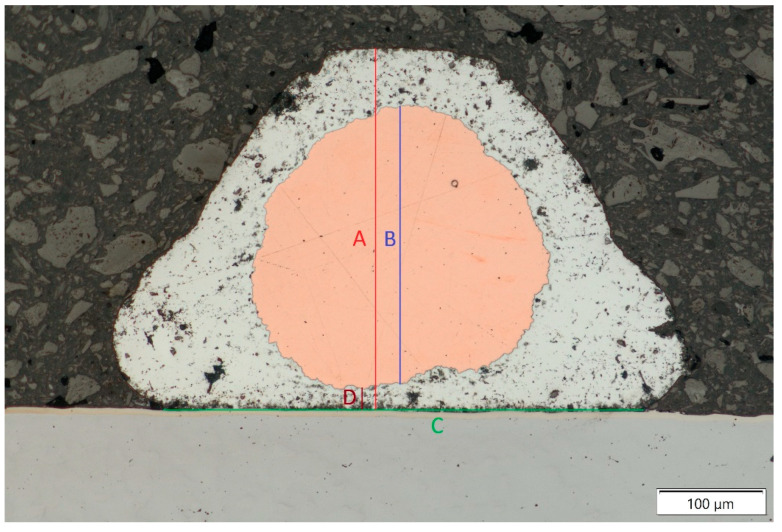
Measurements of the wire bond cross-section with 200× magnification.

Wire bonds were measured as in Figure 5 to establish

A- Wire bond height;
B- Copper core height;
C- Width of the connection on the battery surface;
D- Minimum distance between the copper core and the battery cell surface.

The results of those measurements are presented in Table 3.

**Figure 6:**
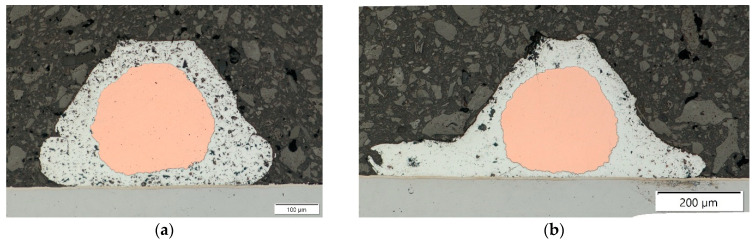
Transversal cross-section made with parameter sets 1 (**a**) and 2 (**b**) in 200× magnification.

**Figure 7:**
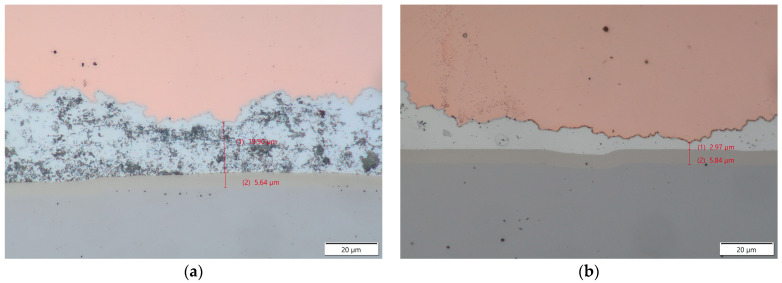
Transversal cross-section made with parameter sets 1 (**a**) and 2 (**b**) in 1000× magnification.

The measurements showed that the wire height as well as the copper core height did not change between the two sets of bonding parameters, as shown in Figure 6a,b. For the more aggressive bonding process that used the second set of parameters, the width of the connection on the battery surface significantly increased, while the distance between the battery surface and the copper core of the wire decreased significantly, as can be seen in Figure 7a,b. This indicated that the aluminum cladding of the wire was pushed out from underneath the copper core to the sides of the wire bond. 

**Figure 8:**
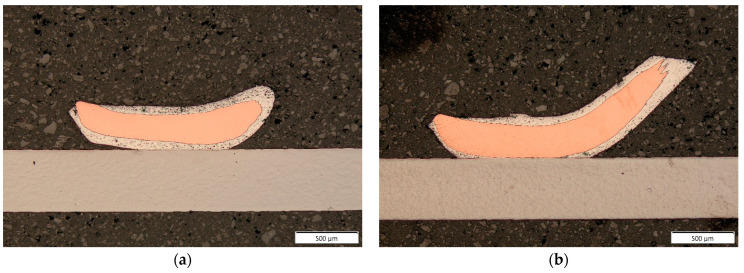
Longitudinal cross-sections made with parameter sets 1 (**a**) and 2 (**b**) in 50× magnification.

Longitudinal cross-sections depicted in Figure 8a,b revealed that for the more aggressive wire bonding process, the distance between the battery surface area and the copper cores was significantly reduced throughout the whole length of the wire bond.

**Figure 9:**
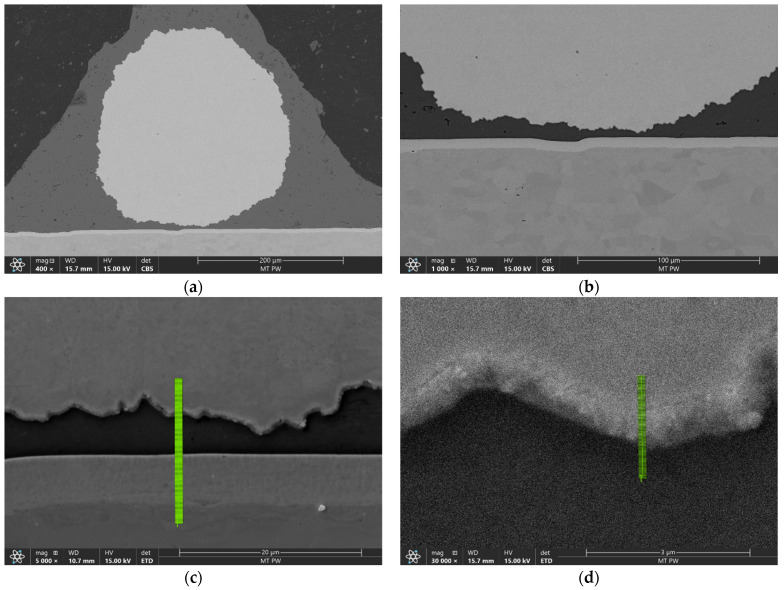
SEM imaging of the transversal cross-section of the bond made with bonding parameter set 2 in magnifications of 400× (**a**), 1000× (**b**), 5000× with EDS line (**c**) and 30,000× with EDS line (**d**).

To better examine the behavior of the materials near the joint interface area, SEM was used. Figure 9a,b shows this area for the sample bonded with the more aggressive parameters.

**Figure 10:**
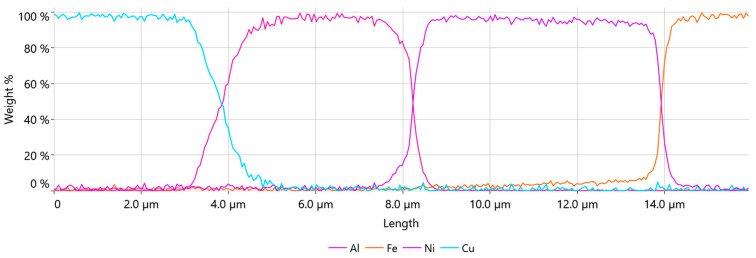
Line EDS result of the wire bond—weight %.

Line EDS was performed to examine the diffusion zone of the materials along the green line shown in Figure 9c. The result is shown in chart form of the weight % distribution of elements in Figure 10.

The transition region between the copper core material and the aluminum cladding was about 1.2 µm wide, while the transition region between the aluminum-clad material and the nickel plating of the battery cell was 0.9 µm wide and was consistent throughout the samples, regardless of the parameter set. This area was much narrower in comparison with our previous study of Heraeus 400 µm AluBond Pure H11 aluminum wire [15]. Since the outer shell material of the wire was almost identical to the H11 wire, this indicated that the copper core introduced changes in the compression behavior of this material.

Since the CucorAl Plus wire introduced a new material interface between the copper core and the aluminum cladding, this area was examined closer with line EDS, as seen in Figure 9d.

**Figure 11:**
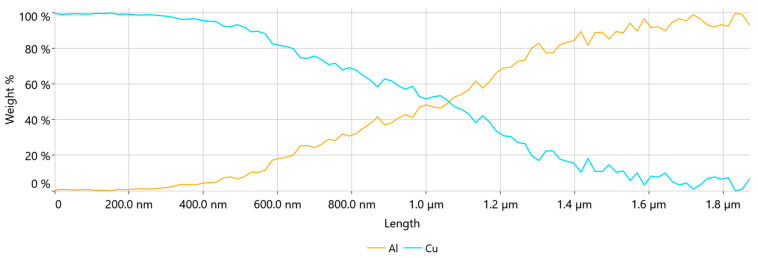
Line EDS result of the wire Cu/Al interface—weight %.

The line EDS results shown in Figure 11 confirmed that the width of the area in which the intermetallic compound was formed was about 1.2 µm. 

**Figure 12:**
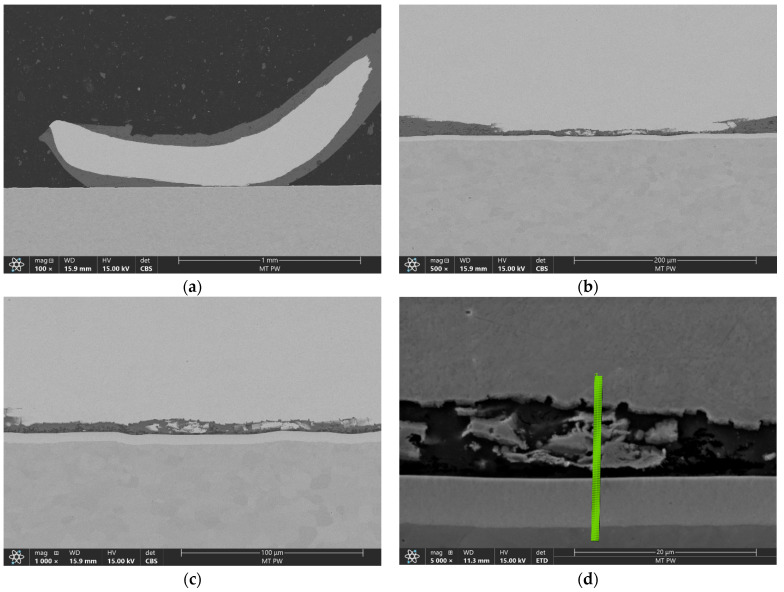
SEM imaging of the longitudinal cross-section of the bond made with bonding parameter set 2 in magnifications of 100× (**a**), 500× (**b**), 1000× (**c**) and 5000× with EDS line (**d**).

To further investigate the formation of intermetallic compounds in the interface area, a sample with the most visible changes in this region was selected. This was one of the samples bonded with the more aggressive bonding parameters and was the only sample with visible mixing that occurred between the aluminum and the copper. SEM images of this sample are presented in Figure 12a–c, while Figure 12d shows the line selected for the line EDS analysis. 

**Figure 13:**
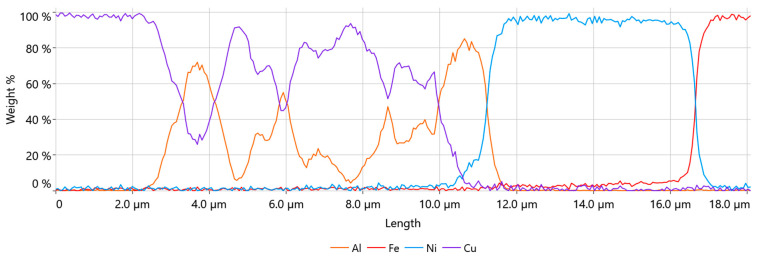
Line EDS results for longitudinal cross-section.

The results of the line EDS shown in Figure 13 prove that for the more aggressive bonding process, the width of the diffusion area could significantly increase by up to 9 µm. Line EDS also revealed that the formation of IMC between Cu and Ni might have occurred in the interface area of the joint.

To examine the mechanical properties of the wire, bonded joint shear testing according to DVS-2811 was performed. 

**Figure 14:**
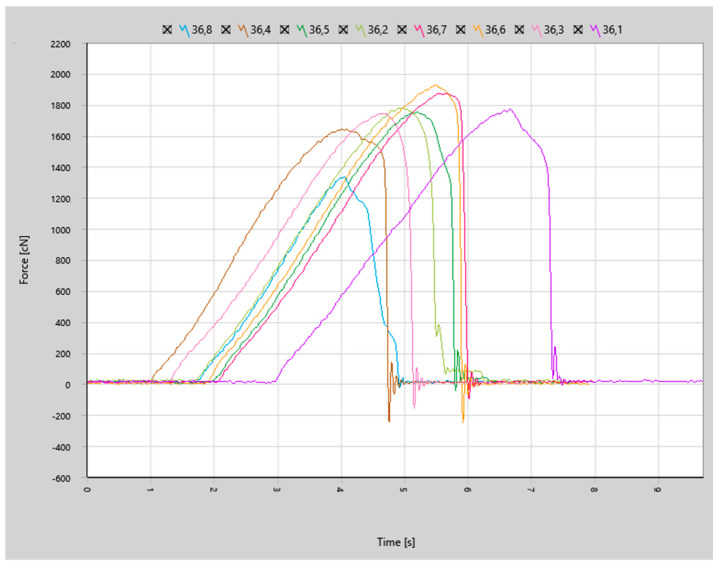
Shear force curves for samples made with bonding parameter set 1.

**Figure 15:**
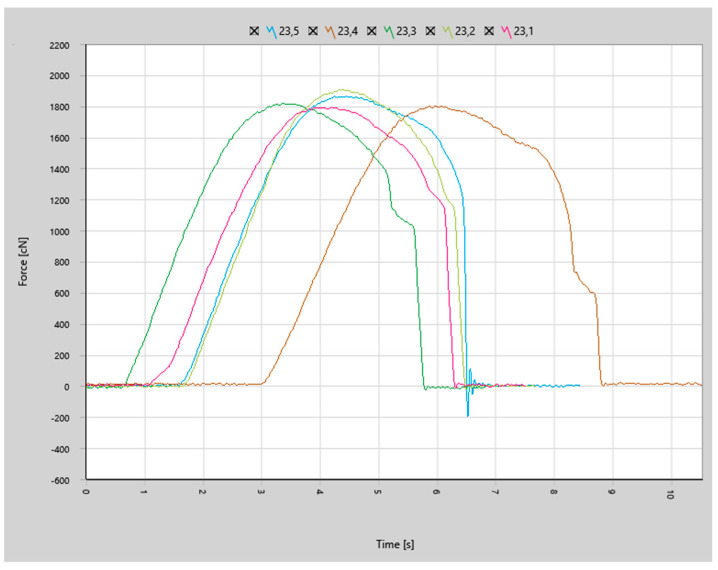
Shear z curves for aluminum wire from previous study [16].

The force curves for the joints bonded with the first set of parameters (less aggressive, identical to the parameters used in our previous study on H11 aluminum wire) depicted in Figure 14 show that the joint failure was more brittle (steeper curve decline after reaching the peak force) in comparison to previous results of bonds made with aluminum wire, as shown in Figure 15.

**Figure 16:**
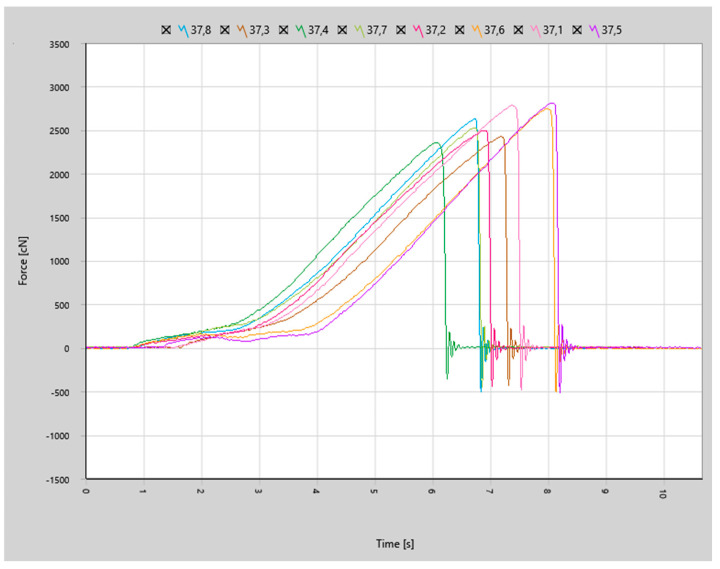
Shear force curves for samples made with bonding parameter set 2.

Force curves for the joints made with more aggressive parameters shown in Figure 16 indicated even more brittle characteristics of joint failure. 

The numeric results of the shear testing are presented in Table 4 and indicate that joints made with Cucor bonding wire were less repeatable but had satisfactory results. The more aggressive bonding parameters had a very positive impact on the shear testing, with a 41% increase in the yield force. The standard deviation of the yield force for the parameter set 2 sample was also below 10%, which was satisfactory.

**Figure 17:**
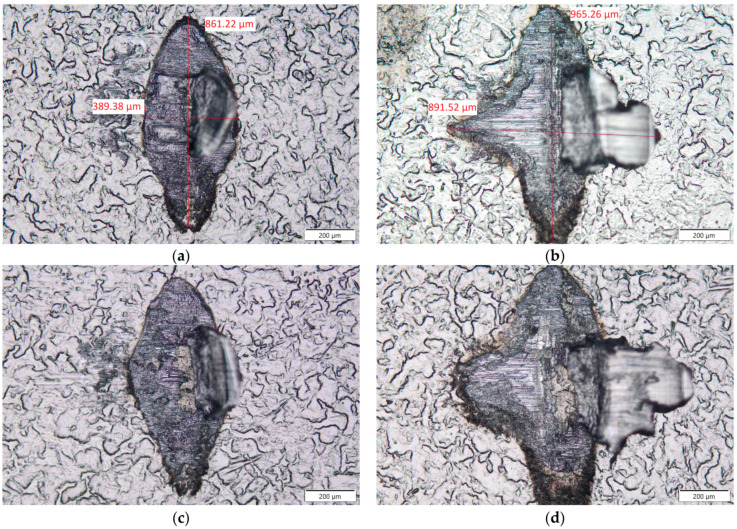
Macroscopic images (50×) of the sheared area for bonds made with parameter set 1 (**a**,**c**) and parameter set 2 (**b**,**d**).

The failure mode of the bonds seen in Figure 17 made with parameter set 1 (Figure 17a,c) and parameter set 2 (Figure 17b,d) shifted from shearing on the tool level observed for H11 Al wire in the previous study to a mix of shearing on the tool level combined with bond liftoff. This indicates that once the sheer tool moving from right to left contacts the copper core of the wire, a liftoff of aluminum cladding at the battery surface level occurs. This mixed mode of failure might be the reason for the more brittle failure of the joints, as indicated by the shear test curves. In some cases, a steel substrate could be seen in the center of the sheared bond, as seen in Figure 17c,d. This indicated a very good connection between the copper core and the outer shell of the wire that was stronger than the joint formed between the outer shell and the battery surface. It should be noted that standards regarding the testing of wire-bonded joints are not intended to be used to judge the failure mode of wires made out of non-homogeneous materials such as two-component wires, so we did not consider the bond liftoff during the shearing of the copper core as a negative test result.

## 4. Discussion

The results of cross-section analyses of the Cucore wire revealed a thin layer of intermetallic compounds in the interface area between the copper core and the aluminum cladding of the wire. This layer was visible under a light microscope and was measured to be about 0.6 µm thick, while line EDS analysis established that the thickness of this layer was about 1.2 µm. Shear tests of the bonds made using this wire revealed that this interface was not a likely point of failure of the joint since the aluminum lifted off with the copper core once the shear tool came into contact with the core in all of the tested samples. This is a very good result proving that the CucorAl Plus wire itself is a stable and well-engineered product that combines two dissimilar metals that are not easily weldable to offer good bendability and high corrosion resistance of high-purity aluminum wire and improved thermal and electrical properties of high-purity copper.

To prove that the Cucor wire retained the good bendability of pure aluminum wire, we used two sets of process parameters. The first parameter set was identical to that used in our previous studies conducted with high-purity H11 aluminum wire. The results were better than we expected for a process developed for different wire materials. The metallurgical cross-sections of joints show no sign of contact between the copper core and the battery case material, which leads us to believe that the joint retained most of the mechanical properties of the aluminum wire established in the previous study. We did observe that the interface thickness between the aluminum cladding of the wire and the battery cell case decreased from 1.6 µm to 0.9 µm. This might be associated with different compression behavior of the aluminum in this area caused by the much harder copper core of the wire, which transferred the force from the sonotrode to the bottom of the wire. The shear testing revealed that the joint retained very similar mechanical properties. It should be noted that the repeatability of the wire bonding process decreased in comparison to the same process conducted with high-purity H-11 aluminum wire but remained at an acceptable level of 10% standard deviation.

The second set of bonding parameters was introduced to achieve a deformation of the wire of about 33% of the wire outer diameter. This is a rule-of-thumb method to establish the process window for pure aluminum wires suggested by wire bonder manufacturers and proven by our team members in the development of numerous industrial projects as well as our previous research. Wire bonding with this second set of parameters led to some very interesting results where, in one of the samples, the copper core came into contact with the battery case, leading to the formation of intermetallic compounds on the interface area of the three materials, which was revealed by scanning microscopy as well as line EDS analysis. We lacked accurate means to further analyze the chemical composition of those compounds and they were detected in only one of the samples. The shear test results indicated a 46% rise in the yield force of the joint. The standard deviation of the shear test results was below 7%, which was higher than that of joints made with high-purity aluminum wire. This is still a very satisfactory result that can be a good argument for the introduction of two-component Cucor wires into the manufacturing process of battery packs.

## 5. Conclusions

This study proves that the wire bonding process can be transferred from aluminum wire to a hybrid copper-core aluminum-clad wire, even without a change in the process parameters. This is advantageous in cases where the current capacity of the aluminum wire is not high enough. Further adjustment of the bonding parameters can significantly improve the process stability and shear resistance of the bonds. Intermetallic compounds on the interface of the copper wire core and the aluminum cladding do not seem to be a weak point of the joint.

## Figures and Tables

**Table 1:** Laser surface cleaning parameters.

Parameter	Value	Description
Speed	2000 mm/s	Speed of the laser beam
Power	40%	Power of the laser (pulse modulation)
Start TC	−100 µs	Time between mirror movement and laser on
Laser off TC	300 µs	Time between mirror stop and laser stop at program end
End TC	300 µs	Time between mirror stop and laser stop at polygon end
Polygon TC	100 µs	Time wait in vector connection point of polygon
Line space	0.01 mm	Distance between consecutive laser lines
Frequency	20 kHz	Laser source switching frequency

**Table 2:** Shear testing parameters.

Parameter	Value	Description
Test distance	800 µm	Shear tool travel during testing
Test speed	100 µm/s	Sheer tool speed
Touchdown force	20 cN	Force required for substrate surface detection
Shear height	40 µm	Height from substrate level at which shear test takes place

**Table 3:** Results of wire bond cross-section measurements.

Bonding Parameter Set	1	2	1	2	1	2	1	2
Measure	A		B		C		D	
Min [µm]	340.94	329.18	254.33	250.94	376.17	436.25	12.58	1.77
Max [µm]	353.24	369.65	268.32	270.87	404.64	543.80	26.45	5.45
Mean [µm]	348.30	350.37	263.38	261.44	388.12	476.12	19.91	3.40

**Table 4:** Shear test results.

	Min	Max	Median	Std. dev.
H11 Al wire [16]	1786.92	1902.05	1814.66	47.95
Parameter set 1	1328.86	1919.67	1756.75	180.47
Parameter set 2	2346.74	2801.96	2569.65	172.06

## Data Availability

The original contributions presented in this study are included in the article; further inquiries can be directed to the corresponding author/s.
